# The Clinical Use of Vernier Acuity: Resolution of the Visual Cortex Is More Than Meets the Eye

**DOI:** 10.3389/fnins.2021.714843

**Published:** 2021-10-05

**Authors:** Monica L. Hu, Lauren N. Ayton, Jasleen K. Jolly

**Affiliations:** ^1^Centre for Eye Research Australia, Royal Victorian Eye and Ear Hospital, Melbourne, VIC, Australia; ^2^Department of Optometry and Vision Sciences, The University of Melbourne, Melbourne, VIC, Australia; ^3^Nuffield Department of Clinical Neurosciences, University of Oxford, Oxford, United Kingdom; ^4^Oxford Eye Hospital, Oxford University Hospitals NHS Foundation Trust, Oxford, United Kingdom

**Keywords:** vernier acuity, hyperacuity, positional acuity, alignment acuity, vernier alignment, vision, visual function

## Abstract

Vernier acuity measures the ability to detect a misalignment or positional offset between visual stimuli, for example between two vertical lines when reading a vernier scale. It is considered a form of visual hyperacuity due to its detectable thresholds being considerably smaller than the diameter of a foveal cone receptor, which limits the spatial resolution of classical visual acuity. Vernier acuity relies heavily on cortical processing and is minimally affected by optical media factors, making it a useful indicator of cortical visual function. Vernier acuity can be measured, usually in seconds of arc, by freely available automated online tools as well as via analysis of steady state visual-evoked potentials, which allows measurement in non- or pre-verbal subjects such as infants. Although not routinely measured in clinical practice, vernier acuity is known to be reduced in amblyopia, glaucoma and retinitis pigmentosa, and has been explored as a measure of retinal or neural visual function in the presence of optical media opacities. Current clinical utility includes a home-based vernier acuity tool, preferential hyperacuity perimetry, which is used for screening for choroidal neovascularisation in age-related macular degeneration. This review will discuss the measurement of vernier acuity, provide a current understanding of its neuro-ophthalmic mechanisms, and finally explore its utility through a clinical lens, along with our recommendations for best practice.

## Introduction

**FIGURE 1:**
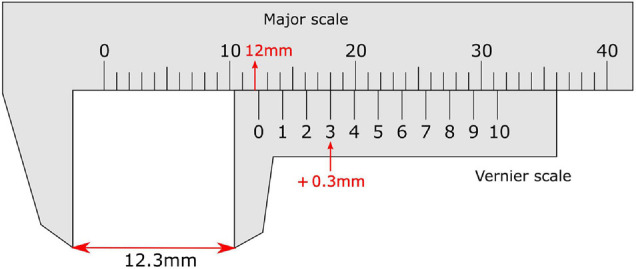
Vernier calliper with a vernier scale on the sliding secondary scale. On the vernier scale the zero line provides the reading before the decimal point (12 mm), while the non-zero line (3) that aligns most closely with a line on the major scale provides the reading after the decimal place. This totals a measurement of 12.3 mm.

The vernier scale, invented in 1631 by the French mathematician Pierre Vernier, allows very precise measurement of length and is read by distinguishing aligned from misaligned vertical lines between adjacent scales (Figure 1). This essentially requires the user to perform a vernier acuity task: to detect small offsets in the alignment between visual objects, in a direction perpendicular to a line joining the objects.

The vernier threshold (smallest detectable offset) for humans is as low as 2 to 5 arcseconds (Westheimer and McKee, 1977b; Westheimer, 1987). Vernier acuity is hence regarded as a type of hyperacuity (Westheimer, 1975), a term that describes visual tasks that have thresholds smaller than the size of a foveal cone (2.5 μm, about 30 s of arc), which limits the classical spatial resolution of the eye. Other examples include stereoscopic acuity (binocular vision), line orientation discrimination, and detection of curvature (Westheimer, 1981).

Vernier acuity is a fascinating and important measure of visual function – particularly cortical visual function – but is infrequently used in clinical practice because it is not well understood. Vernier acuity thresholds can be measured with software that is freely available online with as little equipment as a computer. More complex measurement can be carried out using visual evoked potentials, which require more sophisticated equipment and analysis. Vernier related visual tasks are generally easy for subjects to understand and perform. Measurement of vernier acuity can provide a diagnostic tool that tests a unique aspect of human perception. It has previously been studied in visual disorders such as age-related macular degeneration, glaucoma, and amblyopia. Given its psychophysical characteristics, vernier acuity may also be a useful outcome measure in clinical trials of emerging neuro-ophthalmic treatments, and be useful for more defined diagnostic protocols.

This review aims to bring together broad information on vernier acuity from a clinical perspective, with the purpose of providing guidance on its utility in clinical settings and factors to consider for measuring vernier acuity robustly in a standardised way.

## Measurement of Vernier Acuity

**FIGURE 2:**
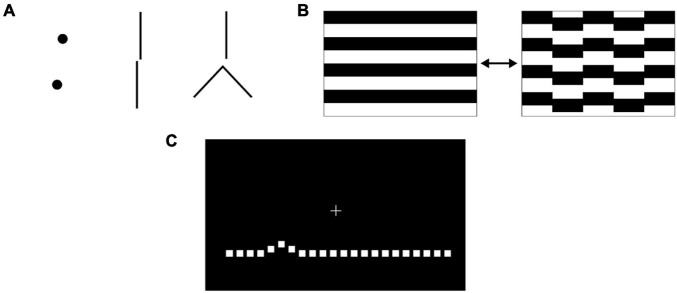
**(A)** Vernier stimuli can consist of pairs of discrete dot-like shapes, lines or other juxtaposed elements where an offset occurs in a direction perpendicular to a line joining the features of interest. **(B)** When measuring vernier acuity with steady state visual evoked potentials, animations that transition between aligned and misaligned stimuli are used. **(C)** Preferential hyperacuity perimetry requires the subject to identify misaligned dots on a computer screen.

Several methods for the measurement of vernier acuity are reported in the literature. Vernier offsets can be detected from stimuli including dots, lines, and gratings (Figure 2), which are usually displayed on a computer screen. Features including the background contrast, luminance, gap size between objects and object sizes can be varied. Psychophysical methods for measuring vernier thresholds rely on behavioural responses based on the judgement of the observer (their visual perception) when viewing the relative positions of these objects. A lower vernier threshold represents better vernier acuity. For example, in a two-alternative, forced-choice (2AFC) paradigm, the observer may be instructed to indicate whether the upper object is offset to the left or the right of the lower reference object. In a three-alternative forced-choice (3AFC) paradigm, the observer may be instructed to distinguish one object-pair that is misaligned (i.e., containing a vernier offset) from two other object-pairs that are aligned (Li et al., 2012). The observer’s response for each trial can be input by either the observer or the examiner. It is thought to be beneficial to give feedback for correct/incorrect responses, so as to maintain the subject’s interest in the task (Enoch et al., 1985). To minimise the effect of perceptual learning, participants can carry out pretraining (Duncan and Boynton, 2003).

Determination of the threshold depends on the method used. When measuring using a staircase procedure, the vernier offset magnitude between stimuli is larger to begin with and is reduced when the observer makes a certain number of correct responses or increased for incorrect responses. Researchers must choose a percentage level of correct responses at which to define the vernier threshold. This level can range from 66 to 85.4% (Enoch et al., 1984b; Li et al., 2012). The number of trials required to complete a staircase can vary from as few as 20 to over 100 (Enoch et al., 1984a; Li et al., 2012), depending on the algorithm used, with 25 trials taking around 3–4 min to complete (Enoch et al., 1985). Another approach reported in the literature is the method of constant stimuli, where stimuli of varying vernier offset size are presented in a random order and the frequency-of-seeing against offset size (psychometric curve) plotted to determine the threshold at a prespecified cut-off (Levi et al., 2000; Li et al., 2000). A constant stimuli procedure involving 300 or more trials was reported to require 20–30 min in experienced observers (Westheimer and McKee, 1977b), although participants in a more recent study only required 2–5 min to complete 105 trials (Latham et al., 2013). Another strategy that can be used is the adjustment method, where the participant must manually position an object on a screen so that it is aligned with one or two other reference objects. In a study with untrained observers using this method, 10 trials were reported to take around 5 min, but due to interindividual variability, the number of trials required to measure a mean vernier threshold with a 10% confidence interval was 100–700 trials (Abbud and Cruz, 2002).

Several key open source computer software programs for the psychophysical measurement of vernier acuity are available online. The vernier acuity module of the Freiburg Acuity Test (FrACT) software^1^ includes an automatic 2AFC staircase with the BestPEST adaptive algorithm on the direction (left or right) of horizontal offset between the position of two vertical lines. The FrACT has been well validated and established in the measurement of acuity in low vision patients (Lange et al., 2009; Jolly et al., 2019). The vernier acuity module provides a valuable adjunct in the battery available. The Psychophysics Toolbox^2^ can be run on Matlab or Octave to display vernier targets and measure vernier thresholds; Psychopy^3^ is a Python-based package that offers similar functions.

Steady state visual evoked potentials (VEPs) can be used as an objective measure of vernier acuity and so are useful in pre-verbal or non-verbal participants, including infants. Electroencephalogram electrodes are placed on the observer’s scalp over the occipital pole [positions O_Z_, O_1_, O_2_; reference at C_Z_ and ground at P_Z_ (Hou et al., 2018)]. A periodic vernier onset/offset animated grating pattern (Figure 2B) is shown to the observer while VEPs are recorded. Spectral analysis of the response demonstrates two components: the odd harmonics of the stimulus frequency (first, third…) correspond to the vernier displacement magnitude, while the even harmonics (second, fourth…) correspond to the motion of the stimulus (Norcia et al., 1999). Plotting the amplitude of the first (1F) harmonic response against the log displacement magnitude yields an approximately linear relationship, and extrapolation of the regression line to a zero response amplitude gives the vernier threshold. Instant transformation of acquired VEP data to VEP vernier acuity can be performed by in-house software (Hou et al., 2018). VEP vernier threshold measurement requires greater technical expertise to conduct compared to psychophysical methods; detailed descriptions of methodology are included in several studies (Norcia et al., 1999; Skoczenski and Norcia, 1999; Hou et al., 2007). In normal as well as amblyopic subjects, vernier acuity measurements made with VEP demonstrate a 1:1 relationship with psychophysical methods of measuring vernier acuity (Hou et al., 2018). VEP measurement of vernier acuity is therefore a valid method that does not require behavioural responses.

Following the COVID-19 pandemic and with rising pressures on clinics for ophthalmic treatments, greater attention is being given to the use of home monitoring to facilitate provision of streamlined care (Jones et al., 2021; Ward et al., 2021). Targeted vernier acuity tools can also be utilised, within the clinic setting or even in a patient’s home, to monitor changes in macular function, particularly important in diseases such as age-related macular degeneration (AMD). Based on detecting the pathological distortion of stimuli that occurs from retinal elevation secondary to choroidal neovascularisation or drusen, preferential hyperacuity perimetry (PHP) can localise and quantify metamorphopsia (Alster et al., 2005). The Foresee PHP (Notal Vision, Tel Aviv, Israel) and its newer home version, ForeseeHome^4^, require the patient to fixate on a central cue on a screen and view a succession of stimuli. Each stimulus consists of a dotted line (Figure 2C), of which some dots deviate from the main axis of the line (an artificial distortion) (Alster et al., 2005; Querques et al., 2011). The patient indicates, either by touch or mouse cursor, where the perceived distortion is. When a pathological distortion is perceived to be larger than the artificial distortion, the patient will preferentially indicate the pathological area. The size of the distortion is varied between trials so that the degree of the pathological distortion can be quantified. Self-monitoring on the ForeseeHome, now approved by the United States Food and Drug Administration for AMD patients at risk of vision loss from choroidal neovascularisation, takes 3 min per eye and assesses the central 14 degrees of a patient’s visual field. As it is a telemonitoring device, the data is automatically sent to the prescribing retinal specialist for review. Although PHP does not output a vernier threshold *per se*, it utilises the principle of a psychophysical vernier task to identify pathology (see section “Retinal Pathologies” for further details and evidence for its use).

## Neuro-Ophthalmic Mechanisms of Vernier Acuity

**FIGURE 3:**
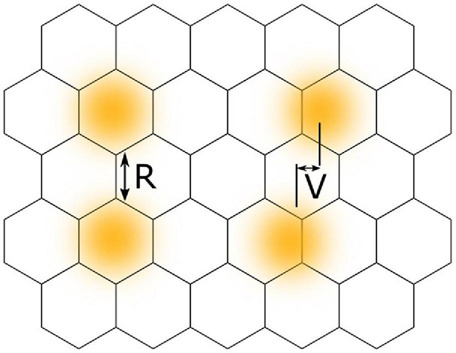
A comparison between the concepts of visual acuity and vernier acuity using a hexagonal mosaic model of retinal photoreceptors. Yellow circles with blurred edges represent areas of retinal illumination. Classical visual acuity, or resolution acuity (R), involves resolving two stimuli as separate and requires a gap in retinal illuminance to be detected by a photoreceptor located between other photoreceptors receiving stimulation. Vernier acuity (V) involves localisation of the difference in spatial positions of two separate stimuli.

Conventional visual acuity, as is usually measured during assessment of visual conditions, relates to the ability of a viewer to resolve two visual stimuli as separate, that is to discriminate their spatial separation. Photoreceptors of the fovea have been shown by microscopy to be arranged approximately in a hexagonal mosaic, each receptor acting as a discrete unit (Westheimer, 2012). For the light from two visual stimuli to be resolved or distinguished as separate, the intervening reduction in light stimulus must be detected by a single receptor that is flanked by two other receptors that receive a greater light signal (Figure 3). Therefore, the resolution limit in the eye as measured by visual acuity matches the spacing between photoreceptors. This forms the basis of visual acuity and is exemplified by the ability to distinguish optotype letters, e.g., “O” from “C” or “P” from “F.” The optical resolution limit, or minimum angle of resolution, corresponds to 1 arcminute of visual angle, or 2 mm when viewed at 6 m. This is a well-studied concept and Westheimer (1987) presents an in-depth description of the limits that diffraction theory imposes on visual acuity. Despite this physical limit, humans can recognise positional offsets of an order of magnitude less than this. This begs the question of the basis of the ability to detect locational differences of less than a cone diameter, beyond the optical and anatomical limits of the eye.

**FIGURE 4:**
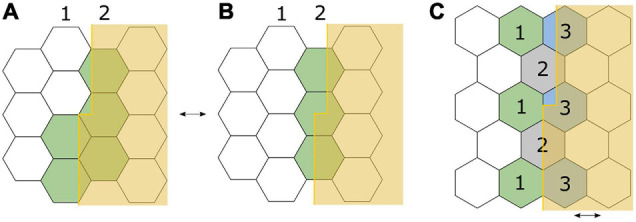
Adaptation of Ewald Hering’s (1899) illustration to demonstrate his eye-movement hypothesis for detection of a small vernier offset, using a hexagonal retinal receptor mosaic model. Each receptor corresponds to an all-or-none “local sign” or “space value.” **(A)** The offset edge lies along two lines of receptors, with the top half exciting only line 2, and bottom half exciting both lines 1 and 2. Excitation is depicted in green. **(B)** The offset edge completely falls along a single line of receptors (line 2) and is therefore perceived as “straight.” Small, repetitive eye movements between **(A,B)** would provide the location difference of the offset lines via higher order averaging (Strasburger et al., 2018). **(C)** In a different orientation, an offset edge lies parallel to the boundaries between receptors. With minute horizontal eye movements across the arrangement, receptors marked 1 and 2 contribute to the location signal of the lower half, and receptors marked 2 and 3 contribute to the location signal of the upper half, thus leading to differential excitation along the edge. Note that on the retinal surface, the edge will not be so sharply demarcated as in this figure; diffraction will result in a steep gradient of illumination.

The exact mechanism underlying the ability of vernier acuity, and other hyperacuities, to surpass this resolution limit of the eye and the retinal elements is still being clarified. Hering (1899) proposed that neural averaging of the signals of receptors along a target (the “local signs”), across minute eye movements, could explain the ability to precisely localise signals (Figure 4; Strasburger et al., 2018). However, as he included the key concept regarding eye movements only in a footnote to his paper, he became widely miscited as having proposed that averaging occurred along the length of the line contour, an idea that in time was disproved by experiments showing that dot and curve stimuli could also act as vernier stimuli. The role of averaging across eye movements was independently put forth again by Andersen and Weymouth (1923) in their ‘retinal mean local sign’ theory. Averill and Weymouth (1925), upon discovering the neglected footnote, subsequently reattributed the origin of this concept to Hering but through their own experiments also contended for the roles of length summation and binocular summation in building the mean local sign. Further knowledge about receptive fields adds to this understanding. V1 is made up of orientation sensitive receptive fields. Both single cell and psychophysical recordings support the sensitivity of hyperacuity threshold to orientation of the target, pointing to the receptive fields being a key component of the underlying mechanism (Swindale and Cynader, 1986; Vogels and Orban, 1990; Fahle and Harris, 1998).

The development of more sensitive experimental techniques, particularly for retinal image stabilisation, has provided further support for the importance of small eye movements in both hyperacuity and conventional visual acuity (Strasburger et al., 2018). Averill and Weymouth (1925) measured a two-fold reduction in vernier thresholds when stimulus exposure time was reduced from 1540 to 30 ms, a time frame chosen to restrict eye movement response, though they were only able to partially control for differences in light flux with their technology at the time. More sensitive methods by Westheimer and McKee (1977a) with full equating of light flux across the exposure period found vernier thresholds relatively unchanged with exposure times as low as 11 ms, suggesting that eye movements are not an absolute limiting factor for vernier acuity (Westheimer, 2018).

Vernier acuity thresholds remain robust to movements when the target is moving up to several degrees per second (Westheimer and McKee, 1975). Performance is reduced if the stimuli are shown asynchronously; even 20–50 ms apart will impair the threshold. Like other visual functions, vernier acuity shows an “oblique effect” where performance is reduced for obliquely oriented stimuli, compared to those aligned with vertical and horizontal meridians (Ludvigh and McKinnon, 1967). This is thought to result from increased positional uncertainty resulting from lower cortical density of neurons tuned to oblique orientations, or increased topographic noise (Saarinen and Levi, 1995). Furthermore, vernier acuity is susceptible to crowding (Westheimer and Hauske, 1975), where masking or interference by nearby stimuli causes reduced performance. This points to a cortical mechanism whereby signals are aggregated and processed from a wider retinal area than that indicated by the stimuli. Flanking lines produce the greatest interference when placed at a distance of 2–4 arcmin from the target stimuli (Levi et al., 1985). Therefore, for accurate and reproducible measurement of vernier acuity it is best to proceed without any crowding markers, while presenting the stimuli synchronously, and ideally in horizontal or vertical meridians. One study found that when obliquely oriented stimuli were presented, participants would attempt to reorient their head, which would interfere with measurement accuracy (Schmid et al., 2018). Overall, it appears from the evidence presented that a cortical mechanism involving processing of ganglion cell impulses, which themselves aggregate the output of many photoreceptors, allows for fine thresholds in the hyperacuity range to be distinguished.

Psychophysical methods, electroencephalography (EEG) and cortical imaging have assisted to elucidate the nature of the cortical mechanisms underlying vernier acuity. Both the primary visual cortex (V1) and extrastriate cortical regions have been implicated in the cortical processing of vernier stimuli. Hou et al. (2017) carried out source imaging studies using functional magnetic resonance imaging (MRI)-informed EEGs to localise sources of vernier and grating acuity in four visual regions: V1, lateral occipital cortex, hV4, and middle temporal cortex. V1 and lateral occipital cortex were the most sensitive cortical areas to vernier displacement stimuli, providing further evidence that detection of vernier acuity involves striate mechanisms. However, grating stimuli (which reflects resolution limits) evoked equal responses in all four regions. Later studies measuring vernier-related activity with steady state VEPs showed a predominant initial response over medial occipital electrodes, with broadly distributed secondary responses occurring later that were consistent with a feedforward pathway originating in the early visual cortex and progressing to higher-order areas (Barzegaran and Norcia, 2020). In contrast, VEPs for letter acuity showed a dominant component over the lateral occipital areas, particularly in the left hemisphere, with later responses at the early visual areas due to feedback. The differences in cortical response topography indicate that while the cortical sources of vernier and letter acuity are distinct, they both undergo processing in the early visual cortex.

Both visual and vernier acuity deteriorate in the periphery compared to central vision. However, the rate at which this happens differs between the 2 modalities, emphasising the difference between these measures of acuity (Levi et al., 1985; Virsu et al., 1987; Wilson, 1991). Whilst visual acuity is restricted by retinal factors and ganglion cell receptive field size, vernier acuity is limited by cortical factors including cortical magnification. When the visual target is scaled to cortical magnification at each eccentricity, vernier acuity performance in the periphery is similar to at the fovea (Levi et al., 1985). The relationship with cortical magnification explains much of the variability between eccentricities, confirming that vernier acuity is a result of higher-level processing. However, cortical magnification does vary between individuals (Horton and Hoyt, 1991). Levi et al. used “perceptive hypercolumns” to describe the psychophysical processing modules of the cortex that subtend a few minutes in the fovea, and found vernier thresholds to be around 1/40th the size of a perceptive hypercolumn. The presence of interfering stimuli within the same or adjacent perceptive hypercolumn reduced vernier performance (Levi et al., 1985). It should be noted that ocular dominance columns, which each span a similar retinal area to perceptive hypercolumns, were not found to be the anatomical site of hyperacuity cortical processing, as hyperacuity thresholds in the test eye were not affected by the absence or presence of interlaced input from the other eye (Westheimer, 1982).

The linear cortical magnification factor in V1, which reflects the cortical spatial sampling of retinal neurons and varies by retinal eccentricity, was measured using functional MRI and demonstrated a correlation between cortical magnification and vernier acuity within observers (Duncan and Boynton, 2003). The computed cortical representation of vernier acuity thresholds was approximately constant across all retinal eccentricities, with a cortical distance of 0.12 mm representing the retinal space occupied by the mean acuity threshold. Due to the consistent cell density in V1, this suggests that a constant number of neurons corresponds to the cortical representation of the mean vernier acuity threshold. Observers with larger overall cortical area in V1 had lower vernier acuity thresholds, and greater changes in the cortical magnification factor with retinal eccentricity were found in observers who also showed greater changes in vernier acuity thresholds with retinal eccentricity. Of the inter-individual variability in vernier acuity thresholds between observers, 21–23% was attributed to cortical topology differences.

Vernier acuity is resistant to contrast and luminance changes at suprathreshold levels (Wehrhahn and Westheimer, 1990; Waugh and Levi, 1993b), with detection reaching its optimum level at a Michelson contrast of 0.22 when background luminance is 860 cd/m^2^. An exponential increase in the vernier threshold at contrast below 0.22 was found. In terms of decimal acuity, vernier acuity is 10-fold that of visual acuity both under photopic and scotopic conditions (Freundlieb et al., 2020). Most studies in the literature test at a Weber contrast of 90% or above, or a Michelson contrast of 80% or above. Studies investigating the relationship between target contrast and target separation using vernier line targets suggest that vernier acuity demonstrates contrast-dependent mechanisms when there is a small separation (2 arcminutes or below) between the targets, whereas larger separations (4 arcminutes or above) are contrast-independent (Waugh and Levi, 1993a). This led to the hypothesis that spatial filters that depend on contrast are involved in the processing of close targets, but not of targets with large separation.

As with other forms of acuity, vernier acuity is improved with binocular summation (Banton and Levi, 1991). Interestingly, when one eye is presented with masking stimuli, the fellow eye will demonstrate elevated vernier thresholds (Mussap and Levi, 1995). This suggests there is an interaction in binocular function beyond summation, and may be reflective of information processing downstream from the initial cortical processing site. Thus for accurate vernier acuity measurement, we recommend each eye be tested monocularly, using a fully black occluder that does not introduce interference to the tested eye.

Training can improve vernier task performance and does not require feedback to be effective. A training schedule of 2000–2500 trials per individual resulted in a 40% reduction in thresholds (McKee and Westheimer, 1978). This is analogous to visual training for amblyopia and stroke. This may have developed evolutionarily due to the role vernier acuity plays in facial recognition, where it was demonstrated that vernier acuity judgments made between eye and mouth positions assist with facial discrimination in synthetic faces (Vesker and Wilson, 2012). Vernier acuity has also been found to play a role in visual word processing. A Chinese study found that vernier acuity was significantly correlated with improved Chinese character form-matching task performance, implying a strong contribution to an early stage of Chinese hierarchical word processing. Grating acuity was not found to have such a correlation (Tan et al., 2018). However, vernier acuity was found to be unaffected in English-speaking subjects with dyslexia (Everatt et al., 1999). This discrepancy in findings may be explained by a difference between character-matching for Chinese characters and reading English words, and the number of components that make up the characters in the different languages. Vernier acuity likely plays a role in other aspects of visual experience, but this has not yet been explored in the literature.

## Vernier Acuity Over the Lifespan

**FIGURE 5:**
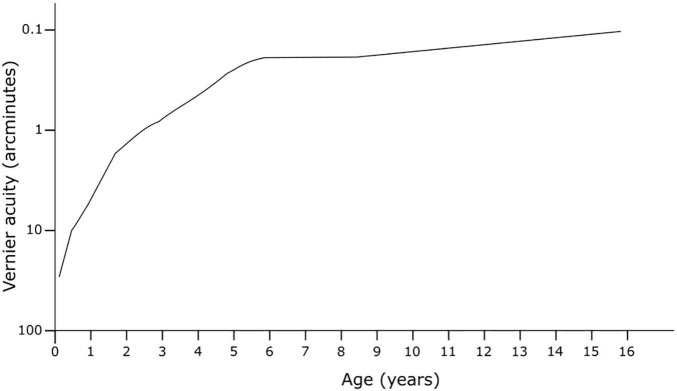
Development of vernier acuity in early life. Adapted from Zanker et al. (1992).

Vernier acuity appears to follow a steeper gradient of improvement across childhood, with maturation likely to occur at around age 6, with further fine-tuning continuing into the teenage years (Figure 5). Earlier methods using preferential looking demonstrated the development of vernier acuity from before six months of age (Manny and Klein, 1984). The early studies found vernier acuity to be poorer than grating acuity in very early life but then superior to grating acuity at around 3 to 4 months, remaining so for the lifespan, a pattern also seen in stereoscopic acuity, another type of hyperacuity (Shimojo et al., 1984). Later studies with more accurate methods using eye tracking with infrared technology, and stationary stimuli to measure both vernier and grating acuity to remove motion as a confounder, indicate that vernier acuity is worse or equal to grating acuity until 4 years of age, at which time it becomes significantly superior (Zanker et al., 1992; Skoczenski and Norcia, 1999). The age of maturity for vernier acuity varies from 6 to 14 years of age (Zanker et al., 1992; Kim et al., 2000). This pattern of development provides support that this is a cortically driven response, as increasing visual input provides opportunity for increased developmental response.

Although early studies (Odom et al., 1989; Lakshminarayanan et al., 1992; Whitaker et al., 1992) had concluded vernier acuity is stable with age in adulthood, this was likely a result of a floor effect when testing younger subjects due to large pixel sizes on screens. Later studies utilising smaller pixel size on higher resolution displays, and therefore enabling a smaller step-size for the vernier offsets, demonstrated that vernier acuity indeed does worsen with age – by about a factor of two above age 60 (Li et al., 2000; Garcia-Suarez et al., 2004). Electrophysiological studies measuring visual evoked potentials confirmed both increased vernier thresholds with age and time required to process vernier stimuli (Li et al., 2001). One suggestion for this effect is a reduction in sampling efficiency with age in the visual cortex due to neuronal changes (Li et al., 2012). This relationship may be disadvantageous for some proposed uses where age may be a confounder, such as for determining potential visual acuity prior to cataract surgery or in long-term studies.

## Current Clinical Utility and Potential Applications

### Cataract

**FIGURE 6:**
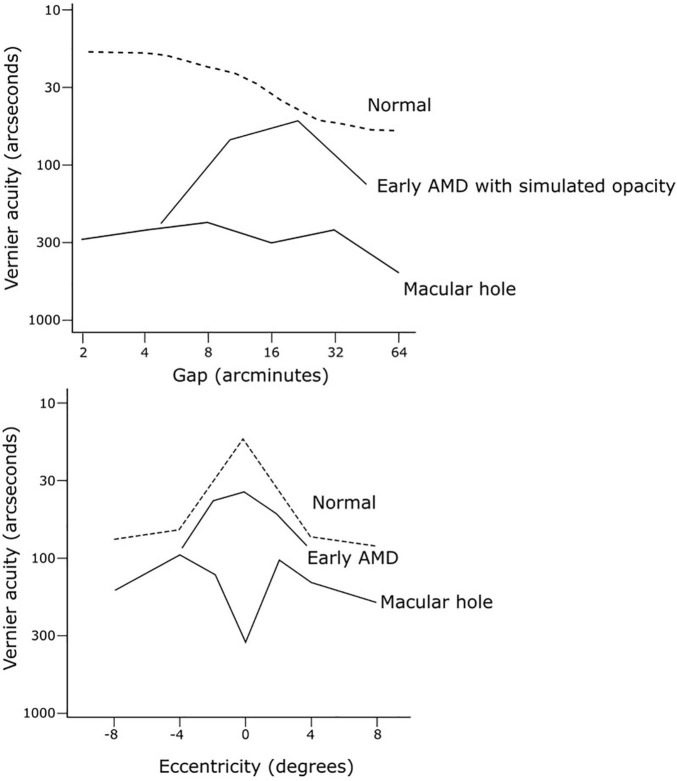
Examples of hyperacuity gap test and hyperacuity perimetry curves from case studies by Essock et al. (1985) to discriminate visual loss due to cataract from that due to retinal pathology. The gap test curve (top) from a subject can be compared to a set of measured curves from patients with cataract resulting in varying levels of visual acuity loss. The hyperacuity perimetry curve in normal vision (bottom, dashed line) is characterised by a central sensitivity peak at the fovea, with flattening (or reversal) of this peak indicating impaired macular function.

Vernier acuity is an indicator of neural visual function. It is highly resistant to retinal image degradation up to a certain point, as well as robust to variations in contrast and luminance, making it an ideal candidate to study neural visual potential in cataract patients (Williams et al., 1984). Conventional visual acuity does not distinguish visual loss due to optical media factors from that caused by retinal or neural factors, and thus may lead to disappointing results post-operatively. In the presence of simulated cataracts by viewing through ground glass, vernier acuity was found to be more resistant to image degradation than resolutional acuity (Williams et al., 1984). This finding was supported on examining patients with real cataracts (Essock et al., 1984). In a series of cases studies, Essock et al. (1985) compared the performance of two vernier acuity tests in differentiating between retinal and optical causes of visual loss (Figure 6). Firstly, they employed a “hyperacuity gap test” that measured vernier thresholds across varying gaps separating dot stimuli. Normally plotting vernier acuity on a log scale against the gap size will produce an inverted U-shape curve, as vernier acuity with dot stimuli is compromised with very large (>6 arcseconds) or very small gap (<2 arcseconds) sizes (Westheimer and McKee, 1977b; Essock et al., 1984). In a normal observer, this curve is shallow with an optimum gap size of 2–4 arcseconds. The characteristics of the curve change with increasing levels of optical degradation from cataract or simulated opacity (ground glass): the optimum gap size (curve peak) increases, overall vernier performance is reduced, and the curve steepness increases (Essock et al., 1985). The case studies demonstrated that the gap test functioned as an indicator of optical quality while being insensitive to retinal dysfunction, and could predict what level of visual acuity loss was accounted for by optical degradation alone. Secondly, they employed a hyperacuity perimetry test that measured vernier performance across retinal eccentricities, which provided information on macular function while being resistant to effects of even severe optical opacities. By comparing the resultant curves from these two tests to a database of curves from patients with normal function or from patients with cataract resulting in varying levels of visual acuity loss (but no other visual condition), they were able to predict whether patients’ reduced visual function was likely primarily due to retinal or optical pathology (Essock et al., 1984). This thus illustrates the utility of this battery of vernier tasks for assessment prior to cataract surgery to predict potential for visual improvement.

### Retinal Pathologies

The PHP (see section “Measurement of Vernier Acuity”) devices are now FDA-approved to monitor for choroidal neovascularisation in age-related macular degeneration (AMD). The Home Monitoring of the Eye (HOME) study was a controlled, randomised clinical trial comparing ForeseeHome use with standard care in 1520 AMD patients (AREDS Home Study Research Group et al., 2014). It demonstrated earlier choroidal neovascularisation detection in patients randomised to home-based device monitoring than in controls, highlighting an important role for vernier acuity in initiating early anti-angiogenic therapy to maximise visual outcomes. A meta-analysis demonstrated a superior pooled sensitivity of PHP (0.85) compared to the Amsler grid (0.78) for screening for the presence of neovascular AMD, although the specificity of PHP (0.87) was lower than that of the Amsler grid (0.97) (Faes et al., 2014). While the current clinical standard for screening for AMD progression is the Amsler grid, it has demonstrated poor efficacy in identifying sight threatening pathology (Fine et al., 1986; Schwartz and Loewenstein, 2015); therefore PHP, with its higher sensitivity, may help improve outcomes for AMD patients despite its lower specificity.

Furthermore, PHP is also useful for monitoring response to anti-angiogenic therapy in neovascular AMD. In a prospective study in 14 AMD patients undergoing a single ranibizumab injection, improvement in Foresee PHP test performance was closely correlated with improvement in macular morphology as measured on spectral domain optical coherence tomography, as well as improvement in best corrected visual acuity (Querques et al., 2011).

Alternative self-monitoring tests using vernier acuity have shown promise for screening for neovascular AMD. An iPad-based Hyperacuity App, which requires the user to complete a task similar to that of the PHP, demonstrated 0.923 sensitivity and 0.615 specificity in identifying choroidal neovascularisation in AMD patients (Chen and Adelman, 2016). Preliminary data from a case-control study on a computer-based Vernier hyperacuity alignment task in diagnosing wet AMD showed 0.75 sensitivity and 0.94 specificity (Schmid et al., 2018). Reduced PHP performance has also been shown in other retinal pathologies such as polypoidal choroidal vasculopathy (Kim et al., 2014) and hydroxychloroquine retinal toxicity (Anderson et al., 2009). Recent work has shown hydroxychloroquine toxicity to be more common than realised (Yusuf et al., 2021) and as such, a screening tool for home monitoring may be of use for early identification of toxicity to reduce disease burden and improve patient outcomes. It is feasible that PHP would be useful to monitor metamorphopsia in other retinal pathologies such as Stargardt disease, but this has not been reported in the literature.

In retinitis pigmentosa, vernier acuity is reduced to a similar degree as letter acuity and grating acuity, suggesting that increased foveal inter-cone spacing is responsible for the reductions in these types of acuities, requiring larger stimulus dimensions in order to perform these tasks to account for the increased grain size of the retinal mosaic (Alexander et al., 1992).

### Glaucoma

Vernier acuity has been investigated as a proxy measure for the change in receptive fields due to ganglion cell loss in glaucoma. Piltz et al. (1993) and McKendrick et al. (2002) found vernier acuity to be an earlier marker than conventional visual acuity or visual field loss, which would indeed support this hypothesis. Mean vernier thresholds were increased by 64% in glaucomatous eyes and 47% in suspect eyes; however, there was significant overlap between the groups (Piltz et al., 1993). A similar pattern was found by McKendrick et al., and additional testing at varying contrast levels did not provide additional sensitivity. This would suggest that vernier acuity would be most helpful in situations where baseline results are available and relative change can be assessed.

### Amblyopia

Amblyopia is a visual disorder resulting from cortical suppression of visual input from one eye during a critical developmental period in early life. It can be caused by strabismus, anisometropia or visual deprivation. Many visual functions are found to be impaired in amblyopic eyes, such as contrast sensitivity, spatial integration (e.g., contour integration and global orientation discrimination), global motion perception, motion-defined form perception, and stereopsis (Wong, 2012). These abnormalities in visual function, and even in more complex cognitive functions, result from dysfunction spanning various areas, including of early processing in V1 and higher level processing in extrastriate areas (Wong, 2012). In unilateral amblyopia, vernier acuity performance is reduced in the amblyopic eye but the fellow eye does not show deficiency, which is in contrast to other tasks involving contrast sensitivity or spatial integration (Meier and Giaschi, 2017). This is a result of the cortical suppression for spatial information corresponding to the amblyopic eye, which leads to spatial distortions and spatial uncertainty. Therefore, an elevated vernier acuity threshold may alert a clinician to neurological or developmental abnormalities so that further investigation or referrals can be undertaken.

Visual evoked potentials measurements in amblyopic patients show a proportional reduction in both vernier acuity and letter acuity (Hou et al., 2018). As both depend on retinal eccentricity, with steep reductions at greater eccentricity from the fovea, this linkage may be explained by the reduced central visual function in amblyopia that occurs secondary to the loss of high-spatial frequency receptive fields and abnormal binocular inhibitory interaction. Vernier performance in the amblyopic eye is more disrupted in strabismic amblyopes than in anisometropic amblyopes, an effect that is greatly magnified when presenting vernier stimuli of high spatial frequency (Levi and Klein, 1982), alluding to differing underlying neural adaptations occurring in these two forms of amblyopia (Meier and Giaschi, 2017). Anisometropic amblyopia is associated with global spatial suppression, whereas strabismic amblyopia causes more localised suppression of the cortical regions that correspond to retinal areas that do not fuse due to the squint. In demonstration of neural plasticity, vernier acuity in the amblyopic eye can be improved with occlusion therapy in children and with practice of vernier tasks in adults (Levi et al., 1997; Meier and Giaschi, 2017). Perceptual learning from the amblyopic eye due to repetitive practice can even transfer to the fellow eye (Li et al., 2008).

Using both VEP and psychophysical measurements in amblyopic patients, vernier acuity has been found to be a more accurate marker of letter acuity loss than grating acuity (Hou et al., 2018). Hence, vernier acuity may be a more reliable method of identifying or monitoring amblyopia than grating acuity, particularly in pre-verbal subjects.

Consistent with the vital role of cortical processing in vernier tasks, children with cortical visual impairment (bilateral visual impairment resulting from perinatal insult to the visual cortex or optic radiations) demonstrate a more severe deficit in vernier acuity compared to grating acuity (Skoczenski and Good, 2004).

### Other Conditions

Vernier acuity has been investigated in a number of other conditions. Vernier thresholds have been found to be elevated in Down syndrome by a factor of 2.7, indicating that impaired cortical processing contributes to poor optical quality in causing reduced visual function (Little et al., 2009).

Vernier acuity, a measure of V1 performance, is unchanged in migraine patients, indicating that the deficit in global form and motion processing in these patients is not mediated through impaired V1 processing (McKendrick et al., 2006).

Vernier acuity can also be used to assess early stage neural processing performance in other visual pathways. Vernier tasks using specific stimuli such as low contrast or isoluminant blue-yellow coloured objects have been employed to study differences in magnocellular (M) and parvocellular (P) visual pathway functions in patients with psychiatric conditions. Subjects with untreated schizophrenia demonstrated dysfunction in vernier tasks involving stimuli specific to the M pathway (Keri et al., 2004), while subjects with bipolar disorder demonstrated impaired M and P pathway functions only during a depressive state (Keri et al., 2007). Psychophysical studies in conjunction with physiological studies in macaque parafoveal ganglion cells have lent support to a hypothesis that M and P pathways have access to spatial position information used by vernier acuity mechanisms (Sun et al., 2012). It has been argued that vernier acuity itself, however, is not an accurate representation of magnocellular sensitivity as conditions that do not cause magnocellular deficits can caused reduced vernier acuity (Skottun and Skoyles, 2010).

## Barriers to Clinical Use

Although vernier acuity or hyperacuity has been proposed and tested as a diagnostic aid for various ophthalmic conditions, including AMD, amblyopia and glaucoma, it has met with several barriers. The measurement process can be time consuming, requiring many trials to measure mean vernier threshold due to interindividual variability. A study using the method of adjustment required a minimum of 100 to 700 trials to achieve a mean precision of 10% (Abbud and Cruz, 2002). Ten trials were reported to take about 5 min, implying 100 trials would require 50 min. Another study using a staircase method to investigate the effect of positional noise on vernier acuity across age groups, including in elderly subjects, required 60–90 min of measurement time across 1 to 2 sessions to record 400 responses per participant (100 responses each at 4 noise levels) (Li et al., 2012). This necessitates balancing the need for a more accurate result against the practicalities of conducting the test within a time period appropriate for participant concentration, as fatigue will also reduce measurement accuracy. The long attention span required for such a task has been suggested as a reason for the higher thresholds in children (Abbud and Cruz, 2002). Furthermore, training changes performance due to cortical plasticity. Where training is incorporated into a study, the results cannot be compared to naïve observers.

Despite the availability of free tools to measure vernier acuity on common equipment such as desktop or mobile computers, vernier acuity is not in widespread clinical use for assessing visual function and is not well understood in the clinical community. The development of commercially available vernier acuity-based tools with clinical utility in AMD allows for the standardisation of testing across research groups and clinical settings, making vernier acuity more accessible. In addition, standardised tools allow validation of these methods to help us better understand the utility of this technique and expected normative values.

## Best Practice Guidance for Vernier Acuity

### Psychophysical Measurement

In order to provide the most reproducible results we recommend to:

• Use one of the available software test suites which have optimised the staircase paradigms:
  ◦ Freiburg Acuity Test (FrACT) software (see footnote 1).
  ◦ Psychophysics Toolbox (see footnote 2).
  ◦ Psychopy (see footnote 3).
• Test without crowding markers.
• Use a gap size of 4 arcminutes for optimum vernier threshold measurement (Westheimer and McKee, 1977b).
• Present the stimuli synchronously.
• Present the stimuli in horizontal or vertical meridians rather than oblique.
• Use high contrast levels of over 90%.
• Test monocularly using a black occluder over the eye not being tested to avoid binocular interference.

### VEP Measurement

• Sweep VEP method (Almoqbel et al., 2008).
• Square wave grating containing a vernier offset and at a Michelson contrast of 80% (Chen et al., 2005).
• ISCEV standard electrode placement for 3 channel VEPs appear to be sufficient (Hou et al., 2007, 2018).

• Use of asymmetric stimuli, such as pattern onset-offset VEP stimuli (Hou et al., 2017).
• Use of multiple luminance levels.
• Use of the first harmonic for threshold determination. Discrete Fourier analysis applied to the amplitude and phase, ensuring the SNR is ≥3:1 with the phase of the response reaming constant or gradually lagging the stimulus as spatial frequency increases (Skoczenski and Norcia, 1999; Watson et al., 2009).

## Conclusion

Vernier acuity, the archetypal hyperacuity, demonstrates the remarkable ability of cortical processing in the visual system to discriminate small offsets between stimuli at a level surpassing the resolution limits of the retina. This provides a measure that is complementary to the measurement of visual acuity by providing information about a different process along the visual pathway. A wide variety of tools that range in complexity have emerged to measure vernier acuity, with some now in clinical use for the monitoring of macular disease. Investigating vernier acuity as a marker of retinal or cortical function has use in ophthalmic diagnostics, as well as a marker of therapeutic success.

The clinical use of vernier acuity has been limited to date by a poor understanding of the technique by clinicians, and limited accessibility to equipment to facilitate testing. However, the availability of open access software (such as the FrACT) now opens the door for increased uptake of this useful test.

To provide more evidence for the clinical implementation of vernier acuity, there are a number of unanswered questions. Further investigations into the use of EEG estimations of vernier acuity (for non-verbal patients) are warranted. PHP could be a beneficial monitoring tool for retinal disease affecting the macula, such as Stargardt disease and bull’s eye maculopathy during hydroxychloroquine intake; longitudinal studies of this tool would be of interest.

Vernier acuity is a useful tool in the diagnosis and management of neuro-ophthalmic disorders. Development of commercial products, such as ForeseeHome, indicate a likelihood of increased uptake in clinical care in the future.

## Author Contributions

MH and JJ created the initial draft of manuscript. All authors contributed to the writing of the manuscript.

## Author Disclaimer

The views expressed are those of the authors and not necessarily those of the NHS, or the funding agencies. The sponsor and funding organisation had no role in the design or conduct of this research.

## Conflict of Interest

The authors declare that the research was conducted in the absence of any commercial or financial relationships that could be construed as a potential conflict of interest.

## Publisher’s Note

All claims expressed in this article are solely those of the authors and do not necessarily represent those of their affiliated organizations, or those of the publisher, the editors and the reviewers. Any product that may be evaluated in this article, or claim that may be made by its manufacturer, is not guaranteed or endorsed by the publisher.

## Footnotes

1
https://michaelbach.de/fract/

2
http://psychtoolbox.org/

3
https://www.psychopy.org/index.html

4
www.foreseehome.com
